# The overlooked role of a biotin precursor for marine bacteria - desthiobiotin as an escape route for biotin auxotrophy

**DOI:** 10.1038/s41396-022-01304-w

**Published:** 2022-08-13

**Authors:** Gerrit Wienhausen, Stefan Bruns, Sabiha Sultana, Leon Dlugosch, Luna-Agrippina Groon, Heinz Wilkes, Meinhard Simon

**Affiliations:** 1 Institute for Chemistry and Biology of the Marine Environment, University of Oldenburg, Carl von Ossietzky Str. 9-11, D-26129 Oldenburg, Germany; 2 Helmholtz Institute for Functional Marine Biodiversity at the University of Oldenburg (HIFMB), Ammerländer Heerstraße 231, D-26129 Oldenburg, Germany

**Keywords:** Water microbiology, Ecosystem ecology, Marine microbiology

## Abstract

Biotin (vitamin B_7_) is involved in a wide range of essential biochemical reactions and a crucial micronutrient that is vital for many pro- and eukaryotic organisms. The few biotin measurements in the world’s oceans show that availability is subject to strong fluctuations. Numerous marine microorganisms exhibit biotin auxotrophy and therefore rely on supply by other organisms. Desthiobiotin is the primary precursor of biotin and has recently been detected at concentrations similar to biotin in seawater. The last enzymatic reaction in the biotin biosynthetic pathway converts desthiobiotin to biotin via the biotin synthase (BioB). The role of desthiobiotin as a precursor of biotin synthesis in microbial systems, however, is largely unknown. Here we demonstrate experimentally that bacteria can overcome biotin auxotrophy if they retain the *bioB* gene and desthiobiotin is available. A genomic search of 1068 bacteria predicts that the biotin biosynthetic potential varies greatly among different phylogenetic groups and that 20% encode solely *bioB* and thus can potentially overcome biotin auxotrophy. Many *Actino*- and *Alphaproteobacteria* cannot synthesize biotin de novo, but some possess solely *bioB*, whereas the vast majority of *Gammaproteobacteria* and *Flavobacteriia* exhibit the last four crucial biotin synthesis genes. We detected high intra- and extracellular concentrations of the precursor relative to biotin in the prototrophic bacterium, *Vibrio campbellii*, with extracellular desthiobiotin reaching up to 1.09 ± 0.15*10^6^ molecules per cell during exponential growth. Our results provide evidence for the ecological role of desthiobiotin as an escape route to overcome biotin auxotrophy for bacteria in the ocean and presumably in other ecosystems.

## Introduction

**Fig. 1:**
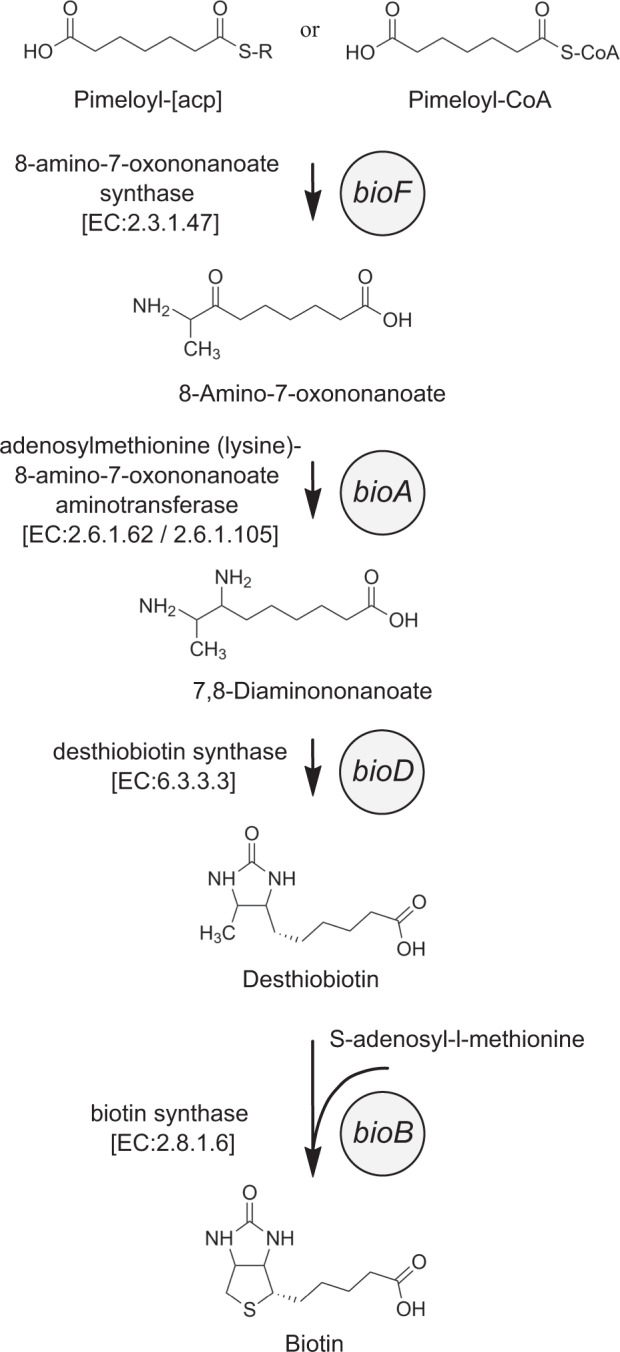
Biotin (vitamin B_7_) biosynthesis pathway. Shown is the biotin biosynthetic pathway as elucidated in bacteria. The individual enzymatic reactions with resulting synthesis products are indicated.

Biotin (vitamin B_7_) is an essential coenzyme involved in numerous biochemical reactions in both prokaryotes and eukaryotes. Biotin-dependent enzymes catalyze carboxylation reactions, such as pyruvate carboxylase, propionyl-CoA carboxylase, acetyl-CoA carboxylase, or β-methylcrotonyl-CoA carboxylase. These indispensable reactions are involved in gluconeogenesis, fatty acid synthesis, and amino acid catabolism [1–3]. Two additional biotin-dependent carboxylases can be found in bacteria, urea amidolyase, which enables bacteria to use urea as a nitrogen source, and geranyl-CoA carboxylase, which is involved in isoprenoid catabolism [4]. The basis for the biotin biosynthesis pathway is the synthesis of pimeloyl-CoA which can be obtained through different metabolic routes [4] followed by the sequential activity of four enzymes, 8-amino-7-oxononanoate synthase (BioF), adenosylmethionine-8-amino-7-oxononanoate aminotransferase (BioA), desthiobiotin synthase (BioD) and biotin synthase (BioB), that convert pimeloyl-CoA into biotin [5, 6]. The final enzymatic reaction involves the insertion of a sulfur atom between the saturated C6 and C9 position of desthiobiotin, which is believed to originate from cysteine [7] (Fig. 1). Current estimates, based on cultivation experiments, suggest that 90 percent of all eukaryotic phytoplankton are biotin prototrophs [8–10]. Despite the importance of biotin and the accessibility of bacterial genomes for the evaluation of metabolic pathways, the biotin biosynthesis potential in marine bacteria has remained largely elusive. In previous studies, only *bioB*, the gene encoding the final conversion of desthiobiotin to biotin, was considered for the classification of the biotin pathway potential of marine bacteria. These analyses suggested that several marine representatives of *Alphaproteobacteria* and *Flavobacteriia* are unable to synthesize biotin, whereas most marine *Gammaproteobacteria* are biotin prototrophs [10]. However, evaluations of the biotin pathway potential of bacterial communities considering all four biotin pathway genes, e.g., in the human gut, show that a large number of bacteria exhibit biotin auxotrophies, yet biotin precursor recovery might play an important role [11].

In the past decades, far-reaching discoveries have been made in the study of B-vitamins in the oceans. Extensive research on the coenzymes thiamine and cobalamin has shown that not only is the exchange of the final cofactor important [12], but also cross-feeding of vitamers (compounds structurally related to a vitamin that include biosynthetic precursors or degradation products) seem to have a hitherto underestimated relevance for the marine B-vitamin cycling [13–17]. Close microbial interactions involving the obligatory exchange of biotin and biosynthetic metabolites between marine plankton have also recently highlighted the importance of biotin for marine microbial communities [18–20]. Research on biotin vitamer cross-feeding in marine ecosystems is still in its infancy, even though first exciting results were obtained many decades ago [21–24]. Several studies demonstrated that fungal and bacterial isolates can overcome their biotin auxotrophy when desthiobiotin is supplied. However, concentrations at which effects were observed were disproportionally high and did not reflect natural conditions [21–24]. Moreover, elevated concentrations of desthiobiotin showed inhibited growth for some bacteria and suppressed the biotin uptake for others [23–25]. What was hidden from scientists at the time (early 1940’s), but is known today, are enzymatic and genetic insights into the biotin biosynthetic pathway to place these findings in an adequate ecological context. In fact, today we know that desthiobiotin is the metabolic precursor of biotin and that its conversion requires the enzyme BioB. Still, cause and frequency of the use of desthiobiotin, as well as its natural sources and availability, are largely unknown. In the few cases where desthiobiotin was measured along a transect in the Atlantic Ocean and at deep-sea hydrothermal vents from the East Pacific Rise, concentrations exceeded partly those of biotin, and the highest concentrations measured to date were around 100 pM at a deep-sea hydrothermal vent [26, 27]. The few dissolved biotin measurements in seawater exhibited strong fluctuations of the final cofactor with values ranging between below the detection limit (<1.2 pM) and up to 700 pM [27–31].

We hypothesize that desthiobiotin has a yet unrecognized important role to overcome biotin auxotrophy in marine microbial communities. Therefore, we asked ourselves how widespread the absence of a biotin pathway, and thus likely biotin auxotrophy, is in marine bacteria and to what extent the availability of desthiobiotin contributes to overcoming auxotrophy. Our goal was to determine natural sources and environmental availability of the currently largely unknown biotin precursor. We further aimed to better understand how bacterial uptake and transformation of desthiobiotin can affect other auxotrophic microorganisms in the environment.

To test whether and how effectively the provision of desthiobiotin enables bacteria to overcome their biotin auxotrophy, we screened several bacteria of the *Roseobacter* group, predicted to encompass biotin-auxotrophic as well as prototrophic organisms [32]. In phytoplankton-bacteria co-cultivation experiments, we investigated whether biotin auxotrophic bacteria with the ability to convert desthiobiotin to biotin can share the final cofactor with ambient microorganisms. Using comparative genomics and metagenomics, we investigated the distribution of biotin pathway genes among marine bacteria. We were able to gain insights into the biotin biosynthesis potential of near-surface prokaryotic communities in a latitudinal transect across the Atlantic Ocean between subantarctic and northern temperature regions. Using high-performance liquid chromatography-coupled tandem mass spectrometry (LC-MS), we identified a natural source of the biotin precursor, desthiobiotin. Our results provide a new insight into the marine biotin cycling, identifying desthiobiotin as an effective alternative for many biotin auxotrophic bacteria suffering from biotin deficiency and highlighting the critical role of B-vitamin vitamers for microbial interactions.

## Material and methods

### Biotin auxotrophy growth experiment

Genome-sequenced bacterial representatives of the *Roseobacter* group were screened for the presence of the biotin biosynthetic pathway. For our growth experiment studies we selected 6 model organisms that either lack the genes encoding the last four enzymatic reactions of the biotin pathway, (*bioF*, *bioA*, *bioD*, *bioB*; *Pacificibacter marinus* DSM 25228, *Sediminimonas qiaohouensis* DSM 21189, *Sulfitobacter* sp. DFL-14) or only possess the gene for the last enzymatic reaction of the biotin pathway (*bioB*; *Celeribacter indicus* DSM 27257, *Roseovarius mucosus* DSM 17069, *Sulfitobacter* sp. EE-36 DSM 11700). Strains were first grown on marine broth (MB)-medium, then washed three times (centrifuged at 5000 g) and resuspended in artificial seawater (ASW)-medium (free of biotin). Cultures were then grown in ASW-medium only supplemented with additions of thiamine (vitamin B_1_), riboflavin (vitamin B_2_), nicotinic acid (vitamin B_3_), pantothenic acid (vitamin B_5_), pyridoxine hydrochloride (vitamin B_6_), and cobalamin (vitamin B_12_; each at 500 pM) to counteract possible other vitamin auxotrophies and supplemented with a substrate mixture (30 mM C), in equal parts (mM C) consisting of glucose, acetate and glutamate. Bacteria were cultured in 10 ml medium in triplicate glass test tubes at pH 8 and 20 °C in the dark on a shaker (100 rpm), with individual additions of desthiobiotin and biotin, 1 nM each, and a negative control with no further addition. Triplicate sterile test tubes containing media and the respective carbon source were used as a control. Growth was monitored by optical density (OD_600_).

As a model organism for a biotin auxotrophic, unicellular, phototrophic eukaryote, the axenic dinoflagellate *Prorocentrum minimum* (CCMP 1329) was grown in charcoal treated seawater (North Sea) medium with minor adjustments. The charcoal treated SW-medium was generated as follows. To the pre-filtered (0.2 µm) SW, 10 g of activated charcoal per liter was added and shaken for 30 min. Then, the charcoal with the bound organic compounds was removed from the SW by filtration with a bottle top filter (Rapid Flow Bottle Top Filter 0.2 µm, Nalgene, Thermo Fisher Scientific, Waltham, MA, USA). The charcoal treated SW was supplemented with NaNO_3_ (882 µM), NaH_2_PO_4_ (36.2 µM) and trace metals as described for the F/2-medium [33], but without the addition of silicate. The medium was flushed with CO_2_ for 1 min before autoclaving. Thiamine (vitamin B_1_), riboflavin (vitamin B_2_), nicotinic acid (vitamin B_3_), pantothenic acid (vitamin B_5_), pyridoxine hydrochloride (vitamin B_6_) and cobalamin (vitamin B_12_; each at 500 pM final concentration) were added to the media to counteract possible other vitamin auxotrophies. In order to maintain a biotin free *P. minimum* culture, the dinoflagellate was transferred 4 times in the charcoal treated SW-medium until we detected no growth in the negative control. The pre-culture for the main experiment of *P. minimum* was spiked with 10 pM biotin to ensure minimal growth of *P. minimum* for inoculation without being a source of biotin contamination. The dinoflagellate *P. minimum* was cultivated in a 12:12 h light-dark cycle illuminated at 70 µE and incubated at 20 °C. Cultures of the main experiment were grown in 40 ml triplicates and spiked with desthiobiotin or biotin (1 nM each) or a negative control without further addition. Triplicate sterile test tubes containing charcoal-treated seawater were run as negative controls. Axenicity of dinoflagellate cultures was verified by staining cultures with Sybr Green and microscopically examining them for bacteria. Growth was monitored by relative fluorescence. To test whether a bacterium that can convert desthiobiotin into biotin supplies another biotin auxotrophic organism with the essential cofactor and in return receives organic carbon, we grew *P. minimum* and *C. indicus* in co-culture under varying experimental conditions. Initially, we grew the co-culture with the addition of desthiobiotin (1 nM) and without further additions. To eliminate the possibility that *C. indicus* is unable to utilize dinoflagellate derived organic carbon and thus is unable to share biotin, in another treatment the co-culture was supplemented with an organic carbon mixture (120 µM C), containing glucose, glutamate and acetate (each substrate at 40 µM C). Further, we supplemented biotin (1 nM) to the bacteria-dinoflagellate co-culture, to ensure that growth of the dinoflagellate was not inhibited by *C. indicus*. Each experiment included a negative control, axenic *P. minimum* grown without biotin addition, and a positive control, *P. minimum* grown with the addition of 1 nM biotin. All treatments were run in triplicates. For all co-cultures treatments, the initial bacterial inoculum was calculated to be at 10^6^ cells ml^−1^.

Samples for bacterial cell counts were collected during the stationary growth phase of *P. minimum*, monitored by relative fluorescence. For bacterial cell counts, samples were fixed with glutardialdehyde (GDA) at a final concentration of 1% and stored at −20 °C until further analysis. Prior to counting by flow cytometry, bacterial cells were detached from the dinoflagellate cells using glass beads (2.3 mm) and ultrasonication (35 °C, 70%, 4 × 5 min, Sonorex, Bandelin, Berlin, Germany), with a short vortex step (2 × 2 s, Vortex Genie2, Scientific Industries Inc., New York, USA) after each ultrasonic interval. This method was a further development of the detachment method described elsewhere [34]. Subsequently, the samples were stained with Sybr Green and counted using a flow cytometer (BD Biosciences Accuri C6; BD Biosciences, Franklin Lakes NJ, USA) as described elsewhere [35].

### Cultivation of *Vibrio campbellii* and North Sea seawater sampling

*Vibrio campbellii* (DSM 19270) was first grown in MB-medium, transferred to autoinducer bioassay (AB)-medium as described elsewhere [36] with slight modifications (Supplementary data set 1) and washed three times with vitamin-free AB-medium before inoculation of the main experiment in AB-medium. Cultures of 1400 ml were supplemented with glutamate (10 mM C) as single carbon source and no vitamins were added. Growth was monitored by OD_600_ and in addition, samples were collected sterilely for bacterial cell enumeration by flow cytometry. Samples for flow cytometry were fixed with 2% GDA, incubated at 4 °C for 30 min and frozen at −20 °C until analysis as previously described [35]. Samples for intra- and extracellular biotin and desthiobiotin determination were withdrawn sterilely during mid- and late-exponential growth phase, as well as early stationary phase. For intracellular biotin and desthiobiotin analyses 50 ml of *V. campbellii* culture were pelleted by centrifugation (5000 g) and frozen at −20 °C until analysis. For extracellular biotin and desthiobiotin analyses 150 ml of culture were filtered through a 0.22 µm bottle top filter (PES bottle-top-filter, Nalgene Thermo Scientific) and frozen at −20 °C until analysis.

Samples for biotin and desthiobiotin analysis were collected during a cruise in July 2019 in the German Bight (North Sea) on RV Senckenberg from 1 m below the sea surface at three different sampling stations (sample 1: 53°34'35“N, 8°10'14“E; sample 2: 53°58‘14”N, 8°36‘55”E; sample 3: 54°45‘38”N, 8°12‘0”E; sample 4: 54°35‘13”N, 6°59‘26”E) using 5 l-Niskin bottles attached to a CTD-rosette (Supplementary Fig. S1A). Water samples were filtered on board (0.22 µm, mixed cellulose esters, Millipore, Billerica, MA, USA) and stored at 4 °C until further analysis.

### Extraction and detection of biotin and desthiobiotin

The extraction of extracellular biotin and desthiobiotin from culture medium and from seawater was performed on a solid phase extraction column (Bond Elut PPL, 1 g, Agilent, Santa Clara, CA, USA), which was conditioned with 20 ml of methanol and 20 ml of H_2_O (adjusted to pH 6 with hydrochloric acid 37%). Filtered North Sea water samples and bacterial exometabolome samples were adjusted to pH 6 with hydrochloric acid (37%) and passed over the column. Ten ml of H_2_O at pH 6 were used to wash remaining salt from the column and the analytes were eluted with 8 ml of methanol. The solvent was evaporated under a stream of nitrogen gas and the dry samples were further processed as previously described [37]. For the extraction of intracellular biotin and desthiobiotin, a modified version of the method described for the analysis of bacterial coenzyme A thioesters was applied [37]. Frozen cell pellets were resuspended in 1 ml of methanol and broken up directly by homogenization in a bead beater. In addition, the extraction was repeated twice with 0.5 ml of methanol and the solvent was evaporated under a stream of nitrogen gas. All other steps were performed as previously described [37]. All filtered extracts were analyzed on a TSQ Quantum AM triple quadrupole mass spectrometer (Thermo Fisher Scientific) with heated electrospray ionization in positive mode (HESI + ). Source parameters were as follows: spray voltage 3000 V, vaporizer temperature 400 °C, transfer tube temperature 340 °C, sheath gas 60 arbitrary units, auxiliary gas 20 arbitrary units. Parameters for selected reaction monitoring mode are listed in the supporting information (Supplementary Table S1). An Ultimate 3000 HPLC (Thermo Fisher Scientific) with a Kinetex Evo C18 column (100 × 2.1 mm, 2.6 µm pore size, Phenomenex, Torrance, CA, USA) was coupled to the mass spectrometer to achieve separation of the individual analytes. The injection volume of every sample was 5 µl and the eluents used were 10 mM ammonium formate (pH 6.0; A) and acetonitrile (B) with the following solvent gradient: 0–13 min 100-75% A; 13–15 min 75–0% A; 15–19 min 0% A; 19–21 min 0–100% A; 21–26 min 100% A. Biotin and desthiobiotin were quantified by external calibration using commercially available standard compounds in combination with recovery experiments under the influence of matrix using seawater and bacterial samples spiked with biotin and desthiobiotin and processed in the same manner. See Supplementary Fig. S2–5 for example chromatograms and MS-fragment spectra with standard signals. In order to draw more accurate conclusions about the number of biotin or desthiobiotin molecules being released by *V. campbellii* at different times during the growth curve, we calculated the detected molecules per cell.

### Detection of genes encoding biotin biosynthesis in genomes of aquatic bacteria

Publicly available genomes were downloaded from the Joint Genome Institute Integrated Microbial Genomes with Expert Review database (accessed 03 November 2021) (JGI/IMG/MER, https://img.jgi.doe.gov/cgi-bin/mer/main.cgi). Prior condition for downloading genomes was ecosystem category “aquatic” and sequencing status “permanent draft” or “completed”. In order to validate completeness of all genomes, the approach we applied was the one described in (ref. [38]) with slight modifications. In short, 55 single housekeeping gene copies were searched for in all genomes [39, 40], whereupon we removed all genomes that had fewer than 53 housekeeping genes, applying a stringent selection. Multiple assigned species were reduced to a single genome, the species with the highest number of housekeeping genes was selected as representative. Strains assigned to a genus but not a species were considered as one group and reduced to one genome, which was also selected according to the highest number of housekeeping genes. We manually checked the list of species for species duplicates.

A total of 1710 genomes (61.6%) was removed from all downloaded genomes, leaving 1068 genomes available for further analysis. The genes of the biotin biosynthesis pathway included in “KEGG orthology (KO) - biotin metabolism” were downloaded into IMG/MER and included the “KEGG function ID” and “EC number” for each enzyme. To verify whether the identified enzymes of biotin metabolism were present in the 1068 genomes, the “Functional profile: function vs. genome” tool was applied. To determine whether the biotin metabolic pathway was present, we were particularly interested in the genes “*bioF*”, “*bioA*”, “*bioD*”, and “*bioB*” (see Fig. 1). Our criterion for assigning bacteria as “biotin prototroph” was the presence of all four genes. If “*bioB*” was present but one or more of the other three genes were absent, the bacteria were considered to be able to recover desthiobiotin “desthiobiotin auxotroph”. If *bioB* was absent as well as other genes, bacteria were classified as “biotin auxotroph”. Detailed information on the assignment of individual bacterial genomes can be found in the Supplementary data set 2. Genes associated to the aforementioned taxa from the Atlantic Ocean Microbiome metagenomes [41] were used to estimate overall abundance of the different biotin biosynthesis phenotypes. In short, Illumina sequence data (European Nucleotide Archive accession number: PRJEB34453) were assembled using metaSPAdes v3.11.1 [42] and genes from contigs larger than 210 bases were predicted using Prodigal [43]. Resulting genes were clustered at 95% sequence identity using USEARCH v10.0.24 (*-cluster_fast*) [44] to obtain a non-redundant set of genes. These non-redundant gene sequences were taxonomically classified using Kaiju v1.6 [45] with the Refseq nr (May 2018) and ProGenomes [46] database including prokaryotic, eukaryotic, and viral sequences. Further methodological details can be found elsewhere [41].

## Results

### Bacterial growth with additions of biotin and desthiobiotin

**Fig. 2:**
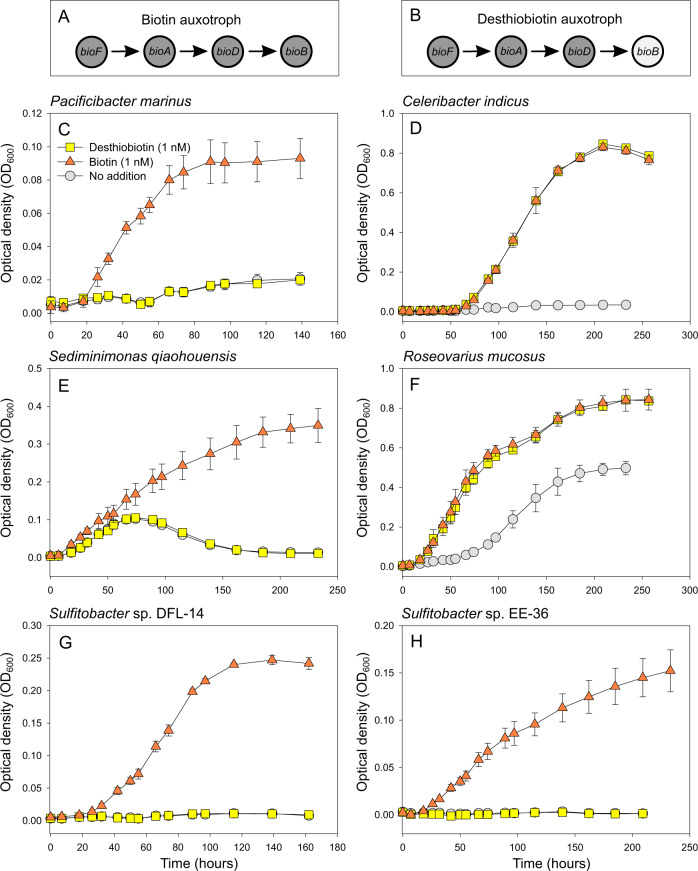
Biotin biosynthesis pathway potential and growth characteristics of strains of the Roseobacter group with additions of biotin or desthiobiotin. **A**, **B** Depicting biotin pathway gene presence of bacterial isolates of the respective growth experiments below (**A** the last four biotin genes are missing; **B** only *bioB* is present). **C**–**H** Growth experiments with the respective additions of biotin (orange, triangle), desthiobiotin (yellow, square) or no additions (gray, circle) for 6 bacterial isolates of the Roseobacter group with different biotin biosynthesis pathway potential (**C**, **E** & **G** biotin auxotroph; **D**, **F** & **H** desthiobiotin auxotroph).

To understand whether the presence of *bioB* alone, but the absence of all preceding genes of the biotin biosynthetic pathway, allows prokaryotes to overcome their biotin auxotrophy, we screened different *Alphaproteobacteria* for their genetic biotin pathway potential. We selected three strains in which the last four biotin pathway genes were absent (*bioF*, *bioA*, *bioD*, *bioB*; Fig. 2A) and three strains in which only the gene encoding the biotin synthase (*bioB*) was present (Fig. 2B).

Two of the tested bacteria lacking all four genes for the biotin pathway, *P. marinus* (Fig. 2C), and *Sulfitobacter* sp. DFL-14 (Fig. 2G) did not grow and *S. qiaohouensis* showed a significantly lower growth yield when desthiobiotin was added as compared to the addition of biotin (Fig. 2E). *Celeribacter indicus* (Fig. 2D), *R. mucosus* (Fig. 2F), and *Sulfitobacter* sp. EE-36 (Fig. 2H), which all possess *bioB*, enabling the last enzymatic reaction to convert desthiobiotin to biotin, exhibited different growth characteristics: *C. indicus* grew with the addition of either biotin or desthiobiotin, but did not grow when neither was supplemented. Growth rate and yield of *C. indicus* was not significantly different when either biotin or desthiobiotin was added at a concentration of 1 nM. Similar results were observed for *R. mucosus* where significantly increased growth was observed with the addition of biotin or desthiobiotin, with no significant difference in growth rate or yield between the two additions (Supplementary Table S2), yet, we also observed weak growth without the addition of either compound. *Sulfitobacter* sp. EE-36, on the other hand, grew only with the addition of biotin, but when desthiobiotin was supplemented, no growth was observed. Since the measurements are based on optical density, we cannot exclude the possibility that in *R. mucosus* it is not the cell number but the cell size that is responsible for the observed difference, nevertheless the addition of desthiobiotin seems to have an influence.

### Growth of *P. minimum* with the addition of biotin and desthiobiotin and in co-culture

We cultured *P. minimum* with and without biotin to determine if this marine dinoflagellate is auxotrophic for biotin. Only the addition of biotin allowed *P. minimum* to grow, as shown by relative fluorescence (Supplementary Fig. S6). To test whether *P. minimum* is able to overcome its biotin auxotrophy by the addition of desthiobiotin, we supplemented *P. minimum* with 1 nM desthiobiotin, but did not observe growth.

Knowing that only the addition of biotin leads to growth of *P. minimum*, we carried out a co-cultivation experiment, growing *P. minimum* with *C. indicus*, which can overcome its biotin auxotrophy by the addition of desthiobiotin (see above, Fig. 2D).

Growth of *P. minimum*, monitored by relative fluorescence, was observed only with the addition of biotin. Supplementation of desthiobiotin to the co-culture did not result in the growth of *P. minimum*. To make sure that it is not the inability of *C. indicus* to utilize the organic carbon sources released by *P. minimum*, we also added substrate to the co-culture at the same time as adding desthiobiotin. Bacterial cell counts measured in the early stationary growth phase prove that, despite increased bacterial growth, there is no release of biotin, which is required for the growth of *P. minimum* (Supplementary Fig. S6).

### Biotin and desthiobiotin concentrations in the *V. campbellii* culture and North Sea samples

**Fig. 3:**
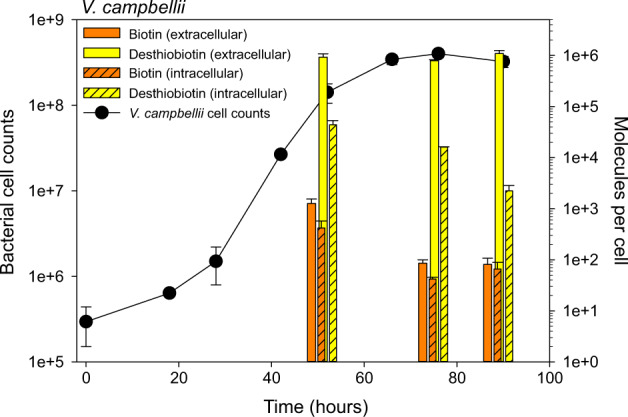
Growth characteristics of *Vibrio campbellii* and detection of extra- and intracellular biotin and desthiobiotin. Growth of *V. campbellii* on glutamate without the addition of B vitamins, monitored by flow cytometer cell counts over time. Bars display extra- (blank bar) and intracellular (striped bar) biotin (orange) and desthiobiotin (yellow) concentrations during exponential, late exponential and late stationary growth phase of *V. campbellii*.

Natural biotin sources and its availability in several marine ecosystems have already been determined [27–30], but who and how desthiobiotin is released and its concentration in large oceanic areas has so far remained largely unexplored [26, 27]. Using mass spectrometry, we measured biotin and desthiobiotin intra- and extracellularly in a culture of *V. campbellii* (biotin prototroph) at three time points during the growth phases and in four seawater samples collected in the North Sea (Fig. 3 & Supplementary Fig. S1). Biotin and desthiobiotin were analysed intracellularly with a recovery of 99% each and extracellularly with a recovery of 30 and 29% and a limit of detection (LOD) of 0.15 pM and 0.03 pM, respectively (Supplementary Table S3). In both intracellular and extracellular samples, the number of biotin molecules detected per cell was relatively low between 42 ± 3.7 and 1267 ± 280.1 molecules per cell. The number of detected extracellular desthiobiotin was much higher, reaching 1.09 ± 0.15*10^6^ molecules per cell in the stationary growth phase of *V. campbellii*. Between intra- and extracellular desthiobiotin concentrations we observed a notable variation. The intracellular number of desthiobiotin molecules per cell reached 4.4 ± 0.91*10^4^ in the mid-exponential phase, 8.1 ± 0.87*10^5^ in the late exponential phase, and 7.2 ± 0.27*10^5^ in the early stationary phase. In contrast, extracellular desthiobiotin molecules per cell continuously decreased from the mid- over the late exponential to the early stationary phase, yielding 9.3 ± 0.15*10^6^, 1.6 ± 0.38*10^4^ and 2.2 ± 0.62*10^3^ molecules per cell.

While biotin was detected in all four North Sea seawater samples examined, desthiobiotin was only detected in one of them, with a concentration of 2.8 ± 0.1 pM. Independent of the sampling locations (estuary, near shore, open sea) biotin concentrations were all at comparative values, ranging between 12.2 ± 0.1 and 19.8 ± 0.6 pM (Supplementary Fig. S1).

### Prokaryotic biotin biosynthetic gene potential

**Fig. 4:**
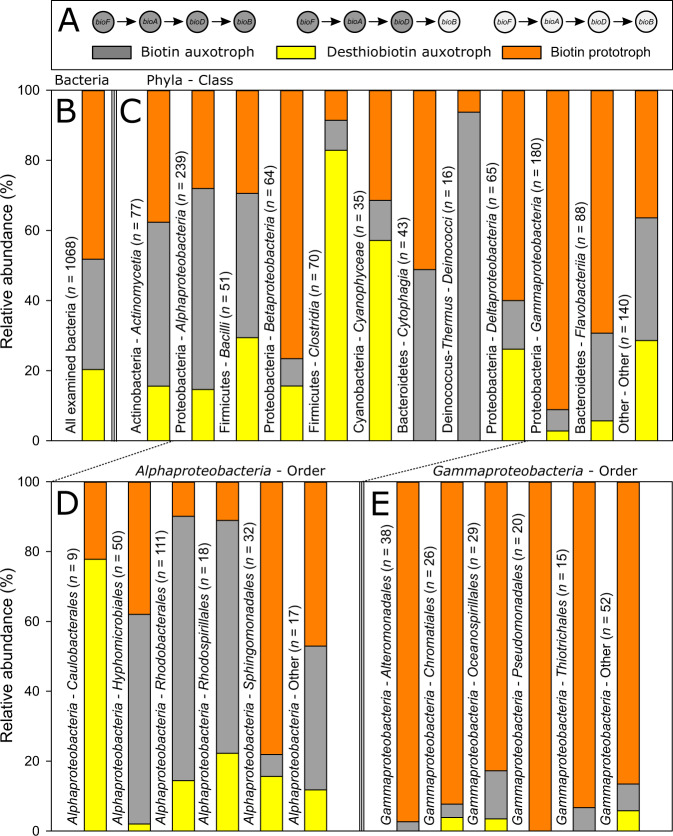
Predicted biotin biosynthesis phenotypes in genomes of aquatic bacteria. **A** Depicted are gene presence for subdivision into biotin biosynthetic phenotypes (biotin auxotroph = gray, desthiobiotin auxotroph = yellow and biotin prototroph = orange) as presented in **B**–**E**. **B**–**E** Biotin biosynthetic genes from 1068 genomes of aquatic bacteria were analysed and classified into biotin biosynthetic phenotypes. Shown is the relative abundance of **B** all bacteria analysed, **C** phyla and classes, **D** orders of *Alphaproteobacteria* and **E** orders of *Gammaproteobacteria*.

To assess which bacteria lack or possess the genetic potential to synthesize biotin, or are likely to overcome a possible auxotrophy by the provision of desthiobiotin, we examined the presence of genes (*bioF*, *bioA*, *bioD*, *bioB*) encoding the last four enzymatic cascade reactions of the biotin pathway (Fig. 1). Considering the 1068 bacterial genomes surveyed, the share of bacteria that cannot synthesize biotin is 51.8% and that of prototrophs 48.2% (Fig. 4B). Of all examined bacteria 20.3% possess at least *bioB* but lack at least one other gene of the biotin pathway and thus require exogenous sources of either biotin or desthiobiotin. In this bacterial group 62.2% encode only the very last gene (*bioB*) of the biotin pathway.

Vast differences exist among the various phylogenetic groups in the ability to synthesize biotin (Fig. 4C). For example, a large share of *Betaproteobacteria* (76.6%), *Gammaproteobacteria* (91.1%), and *Flavobacteria* (69.3%) possess all four genes and thus can likely synthesize biotin *de novo*. The fraction of bacterial genomes that under genetic constraints are biotin auxotrophic but can use desthiobiotin are particularly high in *Clostridia* (82.9%), *Cyanophyceae* (57.1%), and *Bacilli* (29.4%). Highest percentage of true biotin auxotrophs were found in *Alphaproteobacteria* (57.3%), *Deinococci* (93.8%), and *Actinobacteria* (46.8%). Furthermore, we examined *Alpha-* and *Gammaproteobacteria* at the taxonomic level of the order. Especially orders of *Alphaproteobacteria* exhibit significant differences in their biotin biosynthesis potential (Fig. 4D). The SAR11 clade and the majority of *Hyphomicrobiales*, *Rhodobacterales*, and *Rhodospirillales* do not encode the genes encoding biotin biosynthesis or enabling salvation by desthiobiotin. In contrast, the large majority of *Caulobacterales* possesses the ability to recover desthiobiotin and most of the surveyed *Sphingomonadales* possess all genes for biotin synthesis. Biotin pathway potential variations within *Gammaproteobactera* were low, biotin prototrophs dominated all orders (Fig. 4E).

### Pangenome biotin biosynthesis potential in the Atlantic Ocean

To understand the ecosystem implications of our genomic findings of the biotin pathway potential for prokaryotic communities, we linked our genomic analysis to a metagenome reference catalog. Therefore, we carried out a similar analysis in near-surface metagenomes of a latitudinal transect across the Atlantic Ocean from 62 °S to 47 °N covering all biogeographic provinces [41]. The percentage of the prokaryotic community (share of all reads of all genes) for which we were able to account for the biotin pathway potential varied between 2.8% and 11.9%. The high abundance of individual genome sequenced representatives, such as Cyanobacteria in (sub)equatorial provinces, can significantly increase the proportion of known genomes within an analyzed community. Analyzed and represented prokaryotic communities are from a single sampling point and do not reflect temporal or seasonal changes within a community in a given oceanic province.

**Fig. 5:**
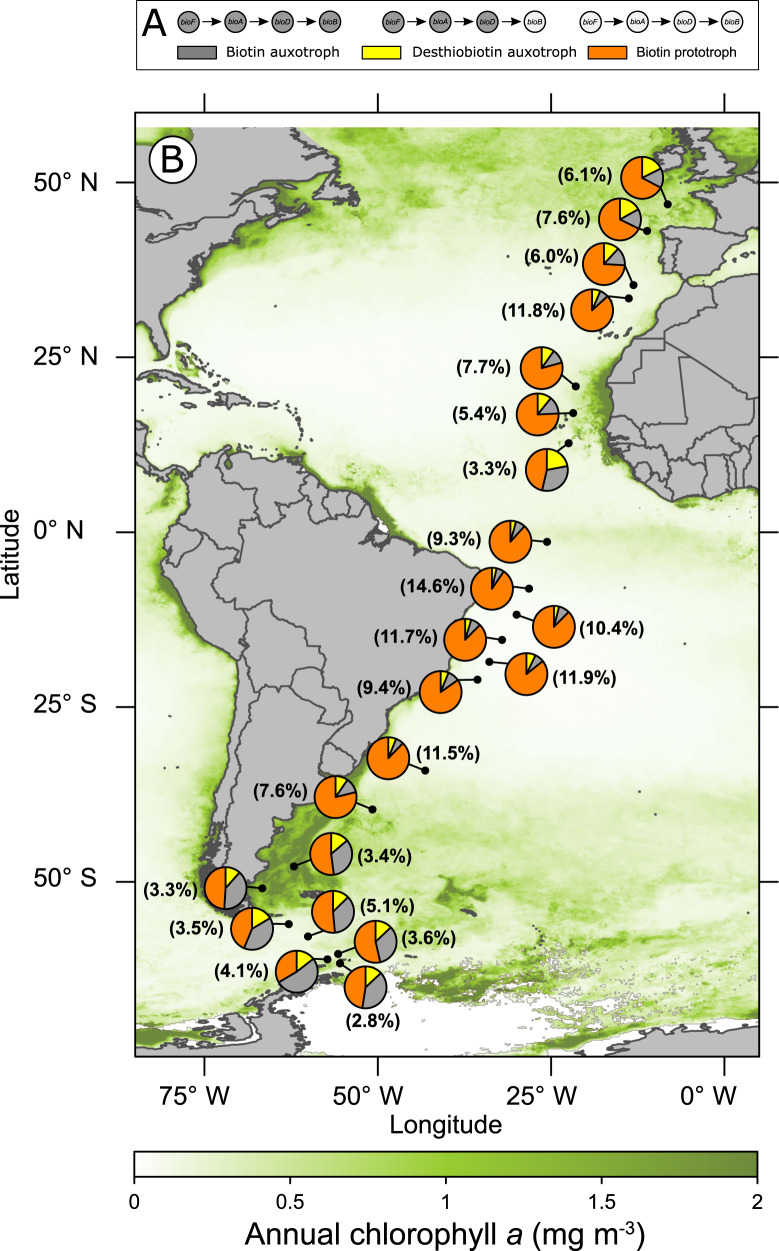
Relative abundance of marine bacteria with varying biotin biosynthesis potential in the Atlantic Ocean. **A** Depicted are gene presence for subdivision into biotin biosynthetic phenotypes (biotin auxotroph = gray, desthiobiotin auxotroph = yellow and biotin prototroph = orange) as presented in **B**. Map depicting 22 stations (black dots) from 62 °S in the Southern Ocean to 47 °N in the North Atlantic, sampled at 20 m depth during a cruise with RV Polarstern. The relative abundance of marine bacteria encoding the last four enzymatic biotin biosynthesis genes (biotin prototroph; orange), bacteria lacking the complete biotin pathway but encoding at least *bioB* (desthiobiotin auxotroph; yellow) and bacteria lacking the biotin biosynthesis pathway genes (biotin auxotroph, gray) is shown in the pie charts of the individual sampling stations. The percentage indicated in each pie chart represents the proportion of genome-sequenced prokaryotes in which the biotin biosynthetic potential of the entire prokaryotic community (identified by the 16 S rRNA gene) was detected. Annual surface chlorophyll *a* concentrations (mg m^−3^) are shown in a green color spectrum.

The fraction of biotin prototrophic bacteria is by far the largest in most regions across the transect and particularly high in the tropics and subtropics (Fig. 5B). However, in the southern temperate and subantarctic region, the share of biotin auxotrophs constitutes at least half of the total community. The share of prokaryotes being able to use desthiobiotin to synthesize biotin and overcome a potential auxotrophy in these regions ranges between 8.5 and 13.1% of the total, equivalent to 14.8–26.1% of the auxotrophic community. In the northern temperate Atlantic, the fraction of biotin prototrophs comprises still at least two-thirds, and the remaining third is mostly equally divided between prokaryotes unable to synthesize biotin *de novo* and those that can overcome their potential auxotrophy by using desthiobiotin (Fig. 5B).

## Discussion

### Biotin biosynthesis potential and its occurrence in the ocean

As early as 1901, biotin was identified as a growth promoting factor [47], followed by its isolation and purification in 1941 [48] and numerous significant discoveries were made thereafter. Several prokaryotic and eukaryotic organisms have been identified as biotin auxotrophs and a growth-promoting effect of biotin vitamers has been observed [21–24, 49]. Since then, biotin has been largely overlooked compared to other well-known and widely investigated B-vitamins, thiamine, and cobalamin, in marine ecosystems [12–17].

Despite major advances in genome-sequencing in recent years, the significance and distribution of biotin auxo- and prototrophy in marine ecosystems remains largely unexplored. Thanks to the study of Sañudo-Wilhelmy et al. (2014) [10], there were first indications that numerous marine bacteria cannot synthesize biotin *de novo*. However, for the determination of biotin synthesizers, the authors did only account for the existence of *bioB*, which catalyzes the last enzymatic reaction in the biotin cascade pathway. In our study we show that of all bacteria that are genomically predicted to be unable to synthesize biotin *de novo*, 39.2% still encode the gene *bioB*. In light of our results, we assume it is likely that the survey by Sañudo-Wilhelmy et al. (2014) falsely identified some bacteria as biotin prototrophs. The results presented here reflect broad variations in the biotin biosynthesis potential between taxonomic groups down to the class level. For example, the majority of *Alphaproteobacteria* does not exhibit a complete biotin pathway. Studies on biotin cross-feeding in co-cultures and defined consortia, where biotin is released from eukaryotic algae and usually taken up by alphaproteobacterial biotin auxotrophic bacteria, support our data [18–20]. Transcriptomic expression of biotin synthesis genes in marine ecosystems from prominent orders of *Alphaproteobacteria*, such as SAR11, SAR116, and *Rhodobacterales* also reflect the dependence of this subclass on biotin [50] and experimentally it has been demonstrated that the abundant member of the SAR11 clade, *Candidatus* Pelagibacter ubique, is a biotin auxotroph [51]. Furthermore, our evaluation shows that the vast majority of both *Gammaproteobacteria* and *Flavobacteria* possess the genetic requirements for biotin synthesis. Again, biotin gene expression confirms that these two taxonomic classes are primary biotin producers in nature [50].

In general, our evaluation suggests that about half of all aquatic bacteria can synthesize biotin *de novo* (Fig. 4B). To shed more light on the actual biotin synthesis potential in marine ecosystems, the abundance of individual representatives should be taken into account. While our analysis covers only between 2.8% to 11.9% of the given prokaryotic community, we can recognize that in subtropical and tropical oceanic provinces the majority of bacteria considered for this study can synthesize biotin *de novo*, whereas in the southern temperate and subpolar zones the share of supposedly biotin auxotrophic bacteria surveyed can rise above 50%. This discovery suggests that prokaryotic communities can differ greatly in their biotin biosynthetic potential, and for a holistic understanding of the biogeochemistry and cycling of biotin, we need to pay more attention to the composition of microbial communities in the future. Whether biotin deficiency can affect planktonic life in the ocean, as has been shown for cobalamin [17, 52], for example, is uncertain at this time. However, we also know for biotin that bacterial growth can be restricted by limited availability of the cofactor even at low pM values in batch cultures [31, 51]. The comparatively few measurements of dissolved biotin in seawater revealed strong variations, ranging from below detection limit (<1.2 pM) to 700 pM [27–31]. Noticeably, especially in the temperate regions with strong seasonal fluctuations, concentrations of biotin in surface waters are in the low picomolar range or below the detection limit [30]. We observed a similar trend in our North Sea water samples, where concentrations reached only 19.8 ± 0.6 pM or even less. Interestingly, our genomic analyses indicate that especially in the regions where the lowest biotin concentrations were measured in the past, the proportion of potentially biotin auxotrophic bacteria is particularly high, up to more than half of the surveyed bacterial community. Even though low concentrations of dissolved biotin do not represent rates of production and consumption but suggest rapid microbial turnover, it appears especially promising to investigate the high latitudes of the northern and southern hemispheres as critical for the effects of biotin availability to marine prokaryotic communities.

### Bacteria overcoming biotin auxotrophy through desthiobiotin uptake

An estimated half of all analyzed aquatic genome-sequenced bacteria are unable to synthesize biotin *de novo* and thus may suffer from biotin auxotrophy and relies on provision by other microorganisms in these ecosystems. A key result presented here is that a large share of potential biotin auxotrophic bacteria encodes the gene for the biotin synthase (*bioB*) which catalyzes the final step of the biotin pathway, and thus can likely overcome their auxotrophy when the biotin precursor desthiobiotin is available. In fact, we have experimentally demonstrated that two bacteria possessing only the biotin synthase can overcome their biotin requirement through the provision of low desthiobiotin concentrations and reach the same growth rate as with the supply of biotin. Whether desthiobiotin enters the cells also by means of the biotin transporters BioMNF, an energy coupling force transporter, or by YigM remains presently unknown [53]. As early as in the 1940s, it was observed that some biotin auxotrophic bacteria could grow with the addition of desthiobiotin [22, 23]. Another two decades later, it was shown for the first time that also individual marine bacteria can overcome their biotin auxotrophy in the presence of desthiobiotin [21]. In contrast to the experiments conducted at that time, we used concentrations that can also be found in the natural marine environment [28] indicating that natural concentrations of desthiobiotin are sufficient to overcome biotin auxotrophy of bacteria. Besides bacteria, also fungi can use desthiobiotin to overcome their biotin auxotrophy [23, 24, 49]. Whether other eukaryotes beyond fungi possess this ability is still unknown, to the best of our knowledge. Research pursuing the ecological importance of these findings was difficult with the state of knowledge at that time and tapered off. On the basis of the genomic evaluation of the biotin pathway potential, we identified a widespread likely ability of various marine bacteria to overcome their potential biotin requirement through desthiobiotin. Thirty-nine percent of all bacteria that are unable to synthesize biotin *de novo* encode a biotin synthase and thus possess the genetic basis to convert desthiobiotin into biotin. However, not all bacteria encoding the gene *bioB* can overcome their biotin requirement under experimental laboratory conditions when desthiobiotin is supplied. This discrepancy between genotype expected phenotypic traits and our experimental observation should be taken into account when interpreting the bacterial biotin metabolic pathway potential. A possible explanation for the observed growth of *R. mucosus* and *S. qiaohouensis* independent of any biotin or desthiobiotin addition could be that there is an alternative, yet unknown route of biotin synthesis or that some bacteria have found a way to bypass a mandatory biotin dependency. The latter scenario is already known for other vitamins, such as cobalamin [54], but to our knowledge has not been considered for biotin so far. However, the share of presumable biotin auxotrophs that encode *bioB* within all biotin auxotrophs of a marine prokaryotic community can account for more than a fifth of the bacterial community analyzed (Fig. 5B). This unexpected large share of bacteria which, with a great probability, are capable of desthiobiotin conversion to biotin challenges our current perspective on the importance of biotin availability and the role of desthiobiotin in the biotin cycle in the ocean.

### Retention of the last step of biotin synthesis

The fact that numerous aquatic bacteria encode the *bioB* gene but have lost the genetic prerequisites for the entire biotin pathway can be well placed in common concepts of microbial ecology. In principle, the observation of gene loss within individual metabolic pathways, as already observed for other B-vitamins [14, 38], appears not to be uncommon and can be explained by genome streamlining theory [55]. Nevertheless, it is remarkable how many bacteria have lost the biotin biosynthesis pathway yet maintaining *bioB*, suggesting that supply by desthiobiotin is well maintained. We can think of two explanations why *de novo* biotin synthesis is lost but the last gene in the biotin synthesis pathway remains present; (i) an enzymatic activity of BioB in a hitherto unknown metabolic reaction; (ii) an evolutionary advantage to be able to convert desthiobiotin into biotin as proposed by the Black Queen hypothesis (BQH) [56, 57]. Although numerous enzymes and enzymatic reactions have been identified, it is very likely that numerous are unknown. For example, *Candidatus* Pelagibacter ubique, a highly abundant member of the SAR11 clade, encodes only the gene *bioA*. It has been proposed that this residual gene of the biotin pathway could play an essential role in the synthesis of pantothenate [51]. Assuming that the encoded enzyme BioB is involved in a hitherto unknown but indispensable metabolic reaction in addition to biotin synthesis, this could provide an explanation as to why so many bacteria possess a streamlined biotin pathway, but still maintain *bioB*. The second explanation describes a scenario in which bacteria benefit from losing genetic traits for the synthesis of certain growth factors, such as vitamins or parts of their biosynthetic pathways, to the extent at which an exogenous supply of these compounds is constantly available. The loss of the biotin pathway, with the exception of *bioB*, leads to lower metabolic cost, as biotin cannot be synthesized de novo, and to a streamlined genome. The downside is that the loss makes auxotrophic bacteria dependent on the availability of the essential micronutrient biotin. Maintaining *bioB* reduces the dependency on biotin in nature and enables the use of desthiobiotin as a second source to overcome a biotin auxotrophy. Another advantage of the genome streamlined desthiobiotin auxotrophic bacteria that should not be neglected, especially for those living in oligotrophic oceanic provinces, is that they save two nitrogen atoms, as these are incorporated upstream of the biotin synthesis. On evolutionary time scales, this could have amplified the loss of the preceding biotin pathway genes.

### Natural sources of desthiobiotin

Retaining a single gene of a pathway, as in the case of *bioB*, may be significant only if the respective required vitamer, desthiobiotin, is available as public good in marine ecosystems. We detect desthiobiotin in one of the four seawater samples that we collected from the North Sea at a concentration of 2.8 ± 0.1 pM. In the Atlantic Ocean, desthiobiotin has been detected at 27 pM, slightly higher than biotin [27] and in samples collected at a hydrothermal vent and seafloor in the Pacific Ocean, concentrations of desthiobiotin were significantly higher than those of biotin, reaching up to 100 pM [26].

Although we know that desthiobiotin occurs in the environment and its molecular mass has been detected by Fourier-transform ion cyclotron resonance MS in the exometabolome of a marine bacterium, its natural sources have remained largely unknown [58]. The experimental results presented here show that a biotin prototrophic bacterium can be a source of desthiobiotin. Despite the fact that *V. campbellii* can synthesize biotin de novo (based on genetic and experimental findings), we were able to detect desthiobiotin intracellularly at a much higher concentration than biotin and at even higher concentrations in its exometabolome. The discrepancy between intracellular and extracellular biotin concentrations might be related to the fact that biotin is covalently bound to enzymes, therefore the cofactor might be intracellularly bound to enzymes and thus not fully captured by our extraction method. The mechanism by which B-vitamins, including biotin, are released is still largely unknown [59]. The chemical structure of desthiobiotin and biotin differs significantly, with biotin having a sulfur-containing tetrahydrothiophene ring fused to a ureido group. Whether the chemical structure affects export or import of biotin or its precursor desthiobiotin remains unclear at this time. Regardless of possible active biotin export mechanisms, an increased leakiness of desthiobiotin compared to biotin could explain its higher extracellular values for *V. campbellii* and thus provide the basis for its widespread uptake and recovery.

Together, these results suggest that desthiobiotin can be produced and released by bacteria and its availability in the ocean may significantly contribute to satisfy biotin auxotrophic microorganisms that retained the biotin synthase. Yet, in addition to bacterial biosynthesis and release of desthiobiotin, another, so far largely unknown mechanism could contribute significantly to the natural source of desthiobiotin. For diatoms, for example, it has been shown that the final enzymatic step (BioB) to convert desthiobiotin to biotin requires iron. When iron is depleted, expression of the biotin synthase *bioB* is greatly reduced [60]. Whether the reduced gene expression upon iron depletion only affects the biotin synthase or a prior enzymatic reaction (BioF, BioA or BioD) has yet not been identified. Inhibition of the biotin synthase without a feedback loop of the biotin cascade pathway, could potentially lead to an excess of desthiobiotin and its subsequent release. In particular, the southern temperate and subantarctic regions of the Atlantic are typical areas where diatoms dominate the phytoplankton community and iron limitation is common [52, 61]. If this is the case, bacteria living in spatial proximity to diatoms could possibly convert desthiobiotin to biotin and thus bridge the negative consequences of iron deficiency on biotin synthesis in diatoms.

### Role of desthiobiotin in the environment

The new data presented here suggest that microbial desthiobiotin cross-feeding contributes significantly to the biotin availability in the ocean. The share of bacteria that possess the genetic basis to convert desthiobiotin into biotin is not equally represented in all taxonomic groups. Bacteria of the Roseobacter group with this ability make up a comparatively large fraction. High abundances of this bacterial group are often found in algal blooms and are known to closely interact with phytoplankton organisms [62, 63]. In order to better understand the impact of desthiobiotin on the marine biotin availability, we co-cultured a biotin auxotrophic bacterium of the Roseobacter group, *C. indicus*, which has been shown to be able to overcome its auxotrophy by the addition of desthiobiotin, with a phototrophic, eukaryotic biotin auxotroph, *P. minimum*. The ability of *C. indicus* to convert desthiobiotin to biotin to overcome its own auxotrophy did not result in the release of biotin to support *P. minimum* in the obligate co-culture. By adding substrate to the co-culture in addition to desthiobiotin, we were able to show that bacterial growth was not inhibited by *P. minimum*. Increased bacterial cell numbers in the stationary phase and growth upon the addition of biotin of both in co-culture also ruled out a reciprocal growth inhibition of either. As our data show, certain bacteria do not necessarily share the desthiobiotin converted into biotin with their environment, even though the heterotrophic bacteria are dependent on the supply of organic carbon compounds, as in our experimental setup. Even if, as in this experiment, there is no direct supply of biotin, it can be assumed that in a natural environment intracellular biotin is made available by sloppy feeding or viral infection.

## Conclusion

We have shown that biotin auxotrophic bacteria that still encode the biotin synthase (BioB) can grow with the provision of low concentrations of the biotin precursor, desthiobiotin. The ability to overcome biotin auxotrophy by the uptake of desthiobiotin appears to be very high among aquatic bacteria and also accounts for a significant proportion in mesotrophic oceanic provinces such as the southern temperate and subantarctic Atlantic. Even though it was already known that desthiobiotin occurs in marine environments, we were able to show that desthiobiotin is also excreted in high concentrations by biotin prototrophic bacteria. Our results suggest that desthiobiotin plays an important role for a large proportion of auxotrophic bacterial communities in the ocean and that the biotin precursor has a previously unknown importance in the marine biotin cycling. Complex microbial biotin-vitamer interactions that go beyond the simple exchange of biotin are a conceivable scenario in the oceans and should be investigated more thoroughly.

## Supplementary information

Supplementary Table 1

Supplementary Table 2

Supplementary Table 3

Supplementary Figure 1

Supplementary Figure 2

Supplementary Figure 3

Supplementary Figure 4

Supplementary Figure 5

Supplementary Figure 6

Supplementary Data 1

Supplementary Data 2

## Data Availability

The datasets generated during the current study are available from the corresponding author on reasonable request.
