# Supplementary material for: The overlooked role of a biotin precursor for marine bacteria - desthiobiotin as an escape route for biotin auxotrophy
Source: ISME J. 2022 Aug 13;16(11):2599–609. doi: 10.1038/s41396-022-01304-w (PMC9561691; doi:10.1038/s41396-022-01304-w)
Supplement: Supplementary file 1 — Supplementary Table 1 [file 41396_2022_1304_MOESM1_ESM.docx]

| **Vitamin** | **Chemical Formula** | **Retention Time (min)** | **Parent Ion *m/z* → Product Ions *m/z* (Collision Energy eV)** | **Tube Lens (V)** |
| --- | --- | --- | --- | --- |
| Biotin (B_7_) | C_10_H_16_N_2_O_3_S | 3.95 | **245.1** → 227.1 (19), 97.1 (41), 123.1 (38) | 110 |
| Desthiobiotin (DB_7_) | C_10_H_18_N_2_O_3_ | 5.15 | **215.1** → 197.2 (17), 179.2 (23), 95.1 (32) | 97 |
