# Supplementary material for: The overlooked role of a biotin precursor for marine bacteria - desthiobiotin as an escape route for biotin auxotrophy
Source: ISME J. 2022 Aug 13;16(11):2599–609. doi: 10.1038/s41396-022-01304-w (PMC9561691; doi:10.1038/s41396-022-01304-w)
Supplement: Supplementary file 2 — Supplementary Table 2 [file 41396_2022_1304_MOESM2_ESM.docx]

| Tested isolate | Doubling time (hours) | | *p*-value |
| --- | --- | --- | --- |
|  | + Biotin | + Desthiobiotin |  |
| *Celeribacter indicus* | 26.25 (± 0.89) | 28.12 (± 0.92) | 0,107 |
| *Roseovarius mucosus* | 18.84 (± 0.33) | 20.15 (± 1.25) | 0,225 |
