# Supplementary material for: The overlooked role of a biotin precursor for marine bacteria - desthiobiotin as an escape route for biotin auxotrophy
Source: ISME J. 2022 Aug 13;16(11):2599–609. doi: 10.1038/s41396-022-01304-w (PMC9561691; doi:10.1038/s41396-022-01304-w)
Supplement: Supplementary file 3 — Supplementary Table 3 [file 41396_2022_1304_MOESM3_ESM.docx]

| **Vitamin** | **Recovery** | | **LOD SPE method** |
| --- | --- | --- | --- |
|  | **SPE method** | **Particulate extraction method** |  |
| Biotin (B_7_) | 30% | 99% | 0.15 pM |
| Desthiobiotin (DB_7_) | 29% | 99% | 0.03 pM |
