# Supplementary figures and images for: The overlooked role of a biotin precursor for marine bacteria - desthiobiotin as an escape route for biotin auxotrophy
Source: ISME J. 2022 Aug 13;16(11):2599–609. doi: 10.1038/s41396-022-01304-w (PMC9561691; doi:10.1038/s41396-022-01304-w)

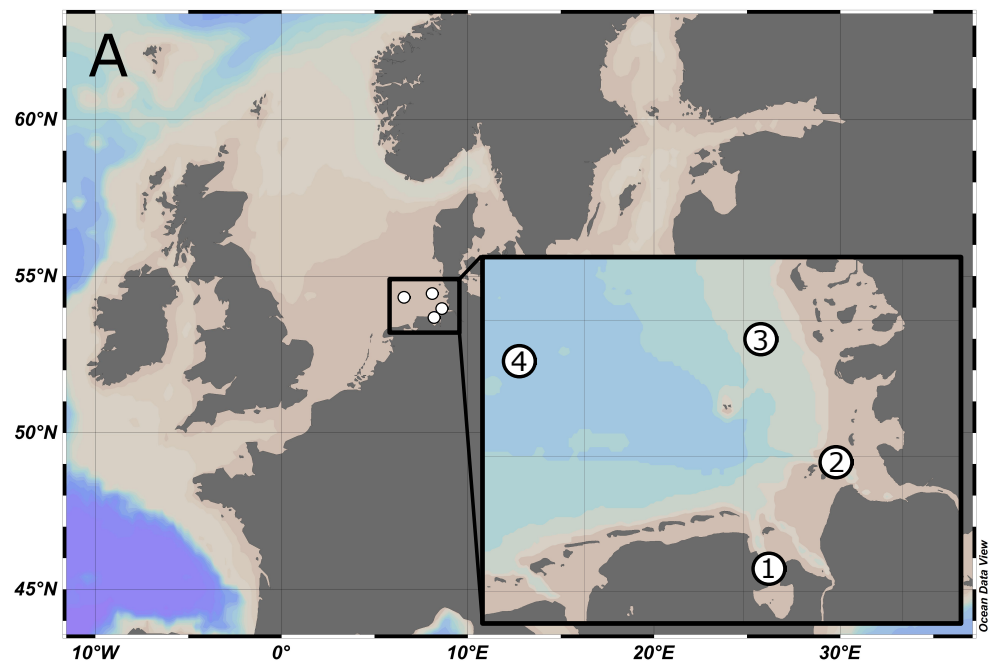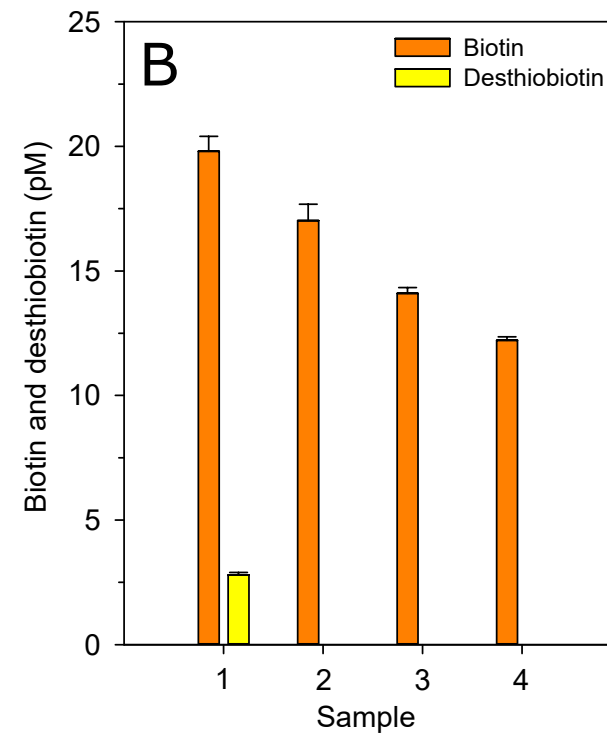

Supplement: Supplementary file 4 — Supplementary Figure 1 [file 41396_2022_1304_MOESM4_ESM.pdf]

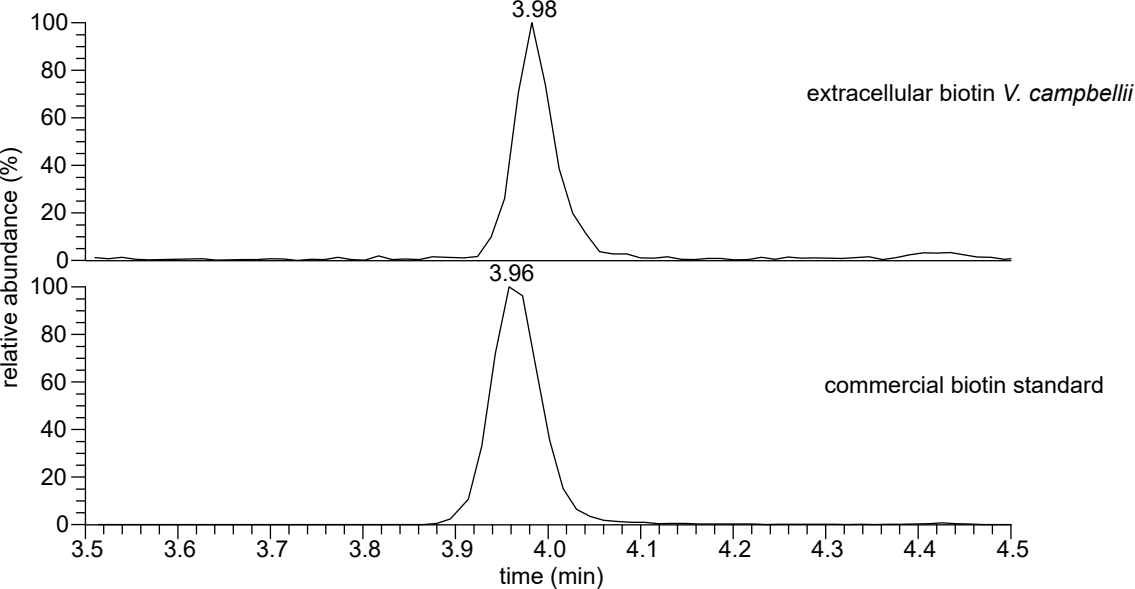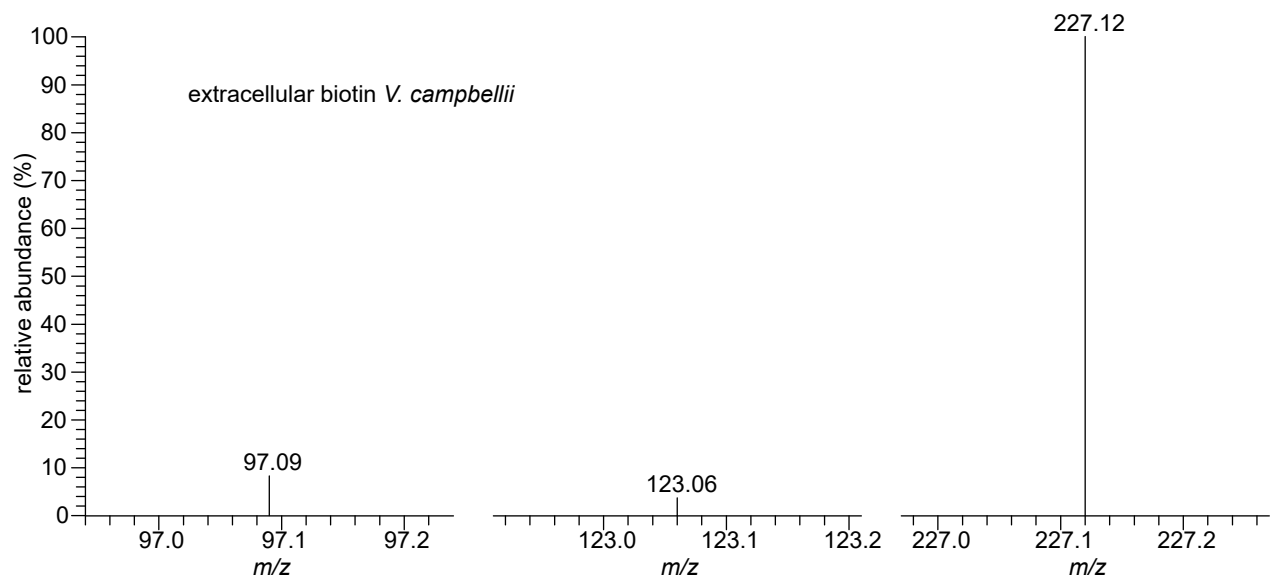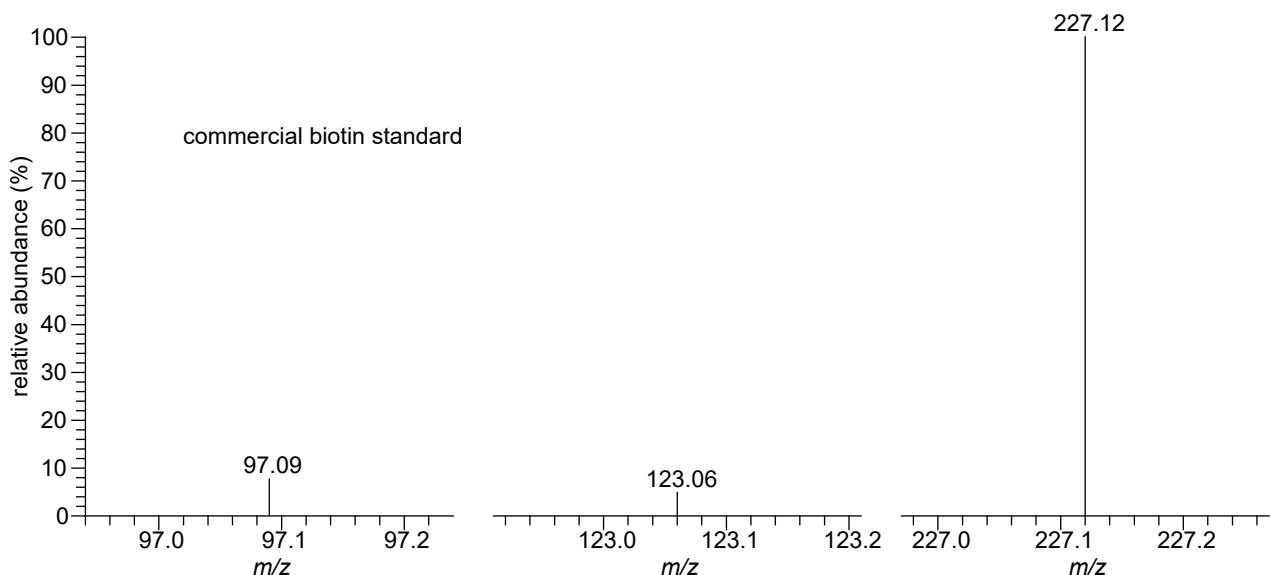

Supplement: Supplementary file 5 — Supplementary Figure 2 [file 41396_2022_1304_MOESM5_ESM.pdf]

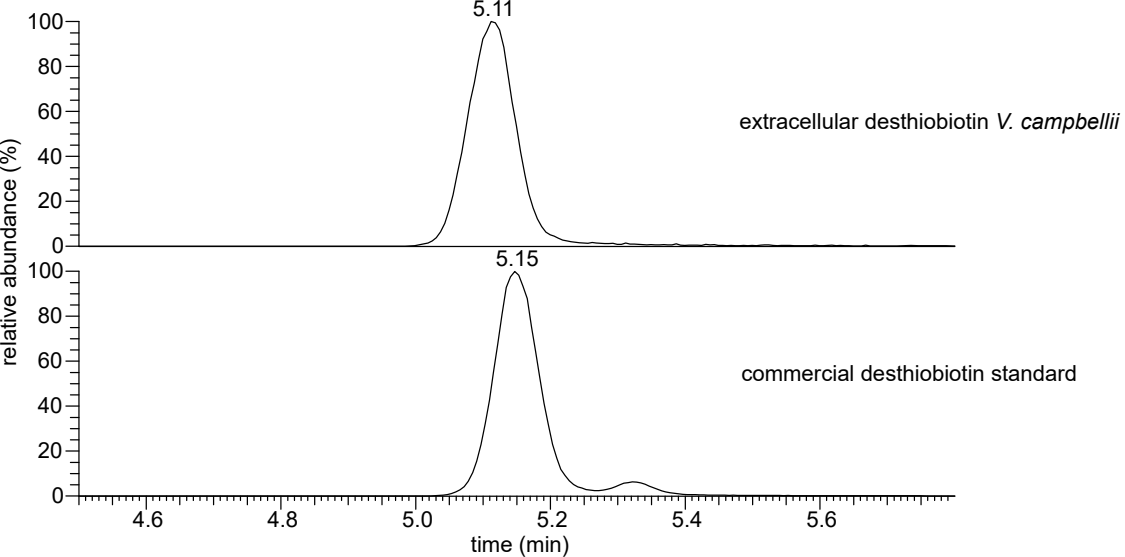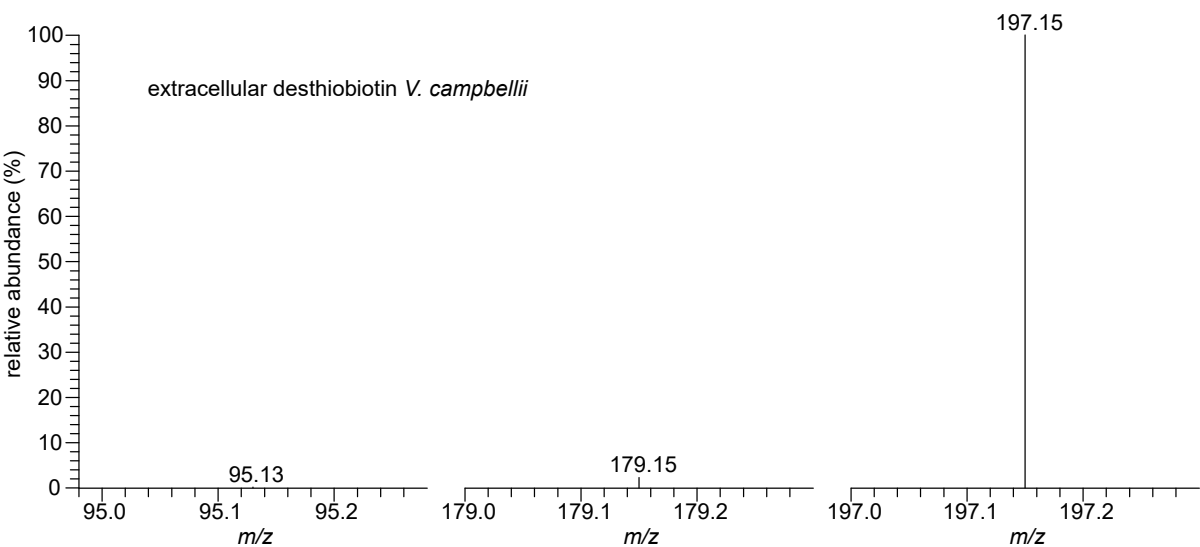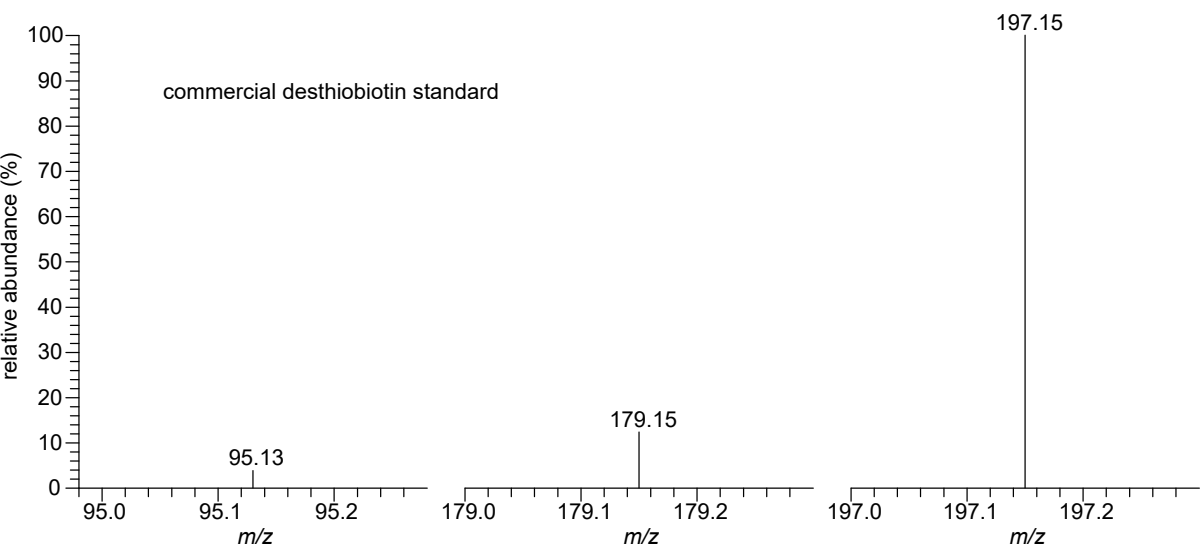

Supplement: Supplementary file 6 — Supplementary Figure 3 [file 41396_2022_1304_MOESM6_ESM.pdf]

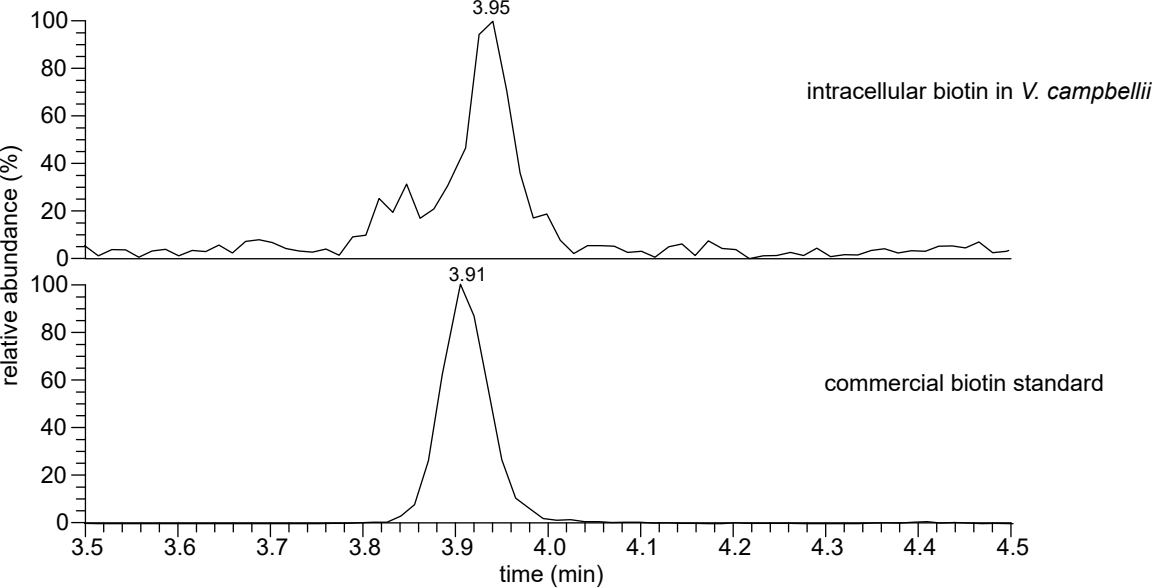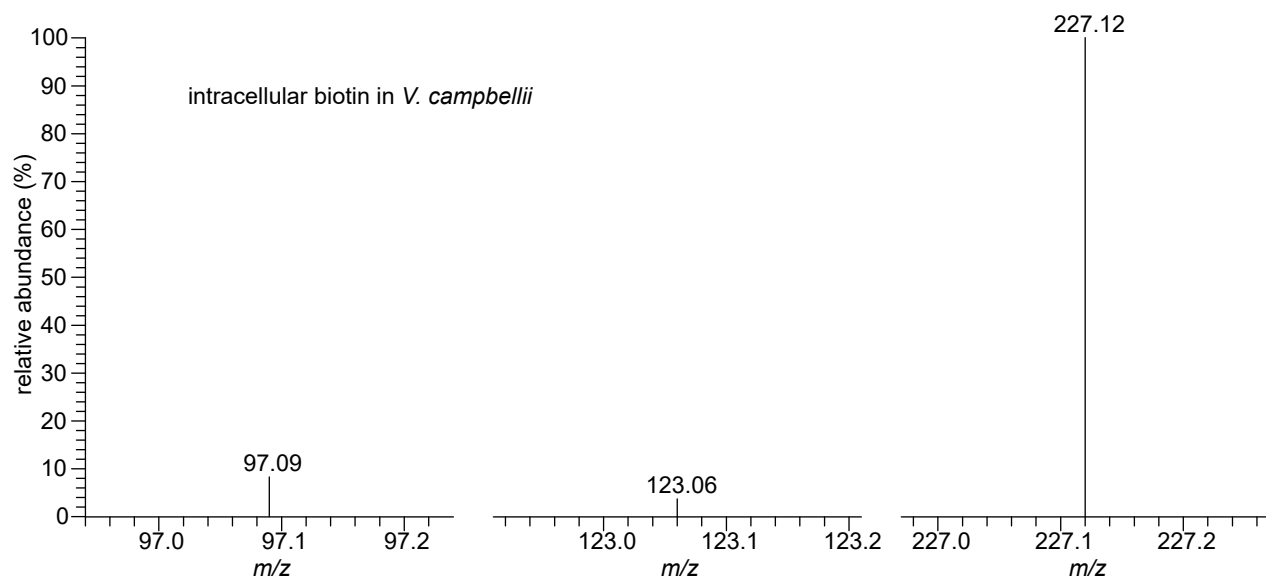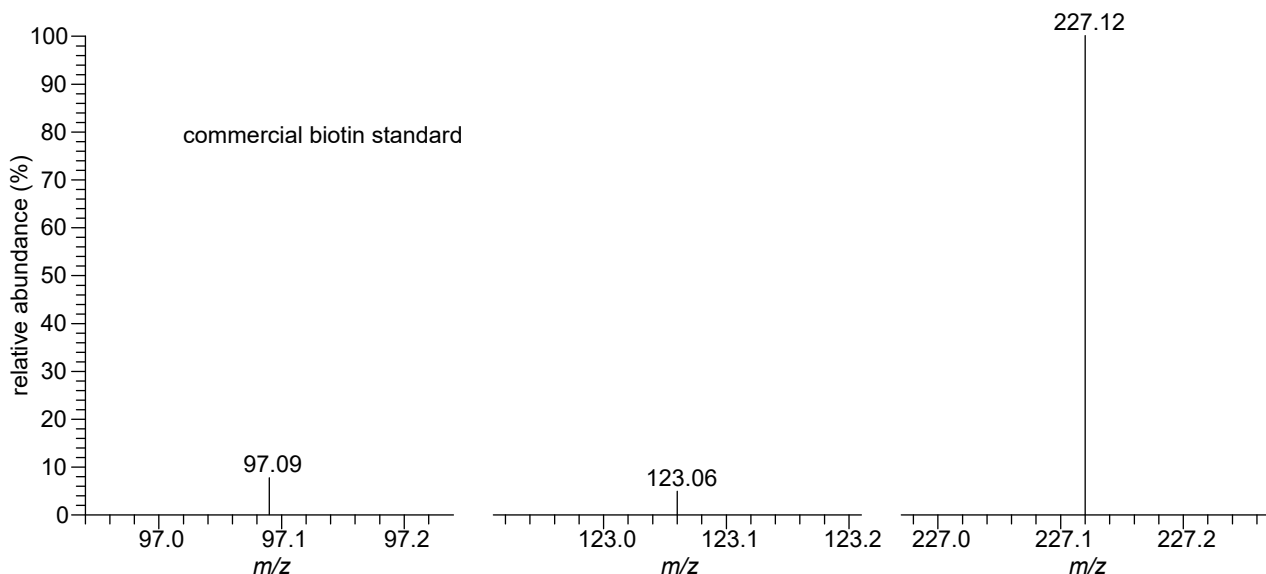

Supplement: Supplementary file 7 — Supplementary Figure 4 [file 41396_2022_1304_MOESM7_ESM.pdf]

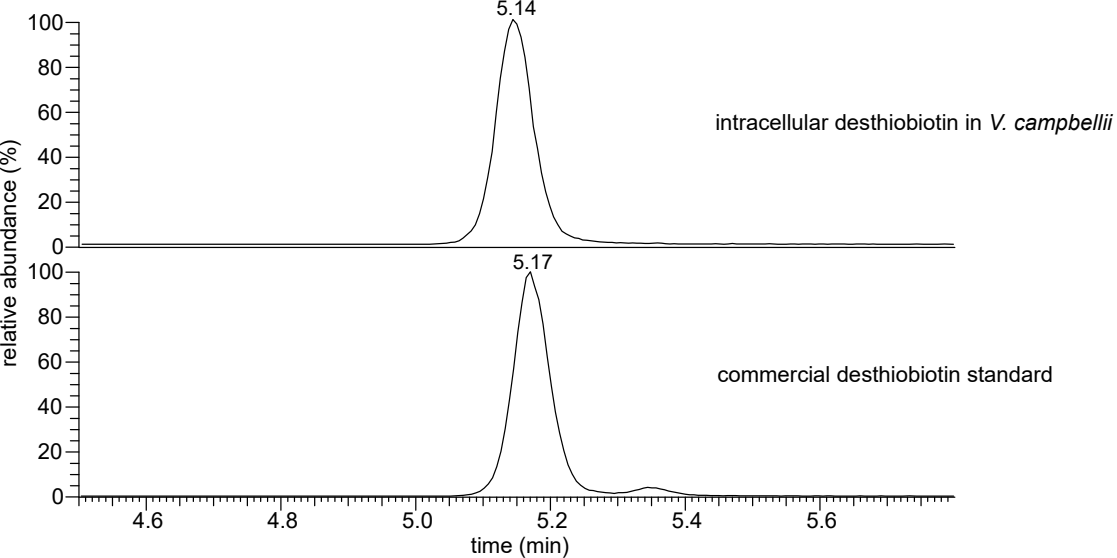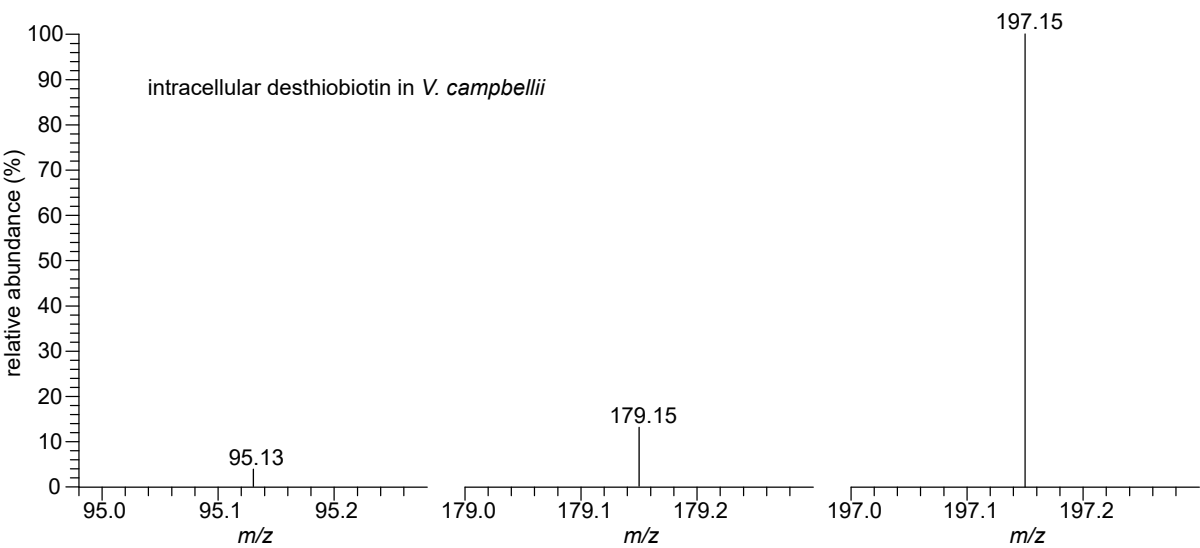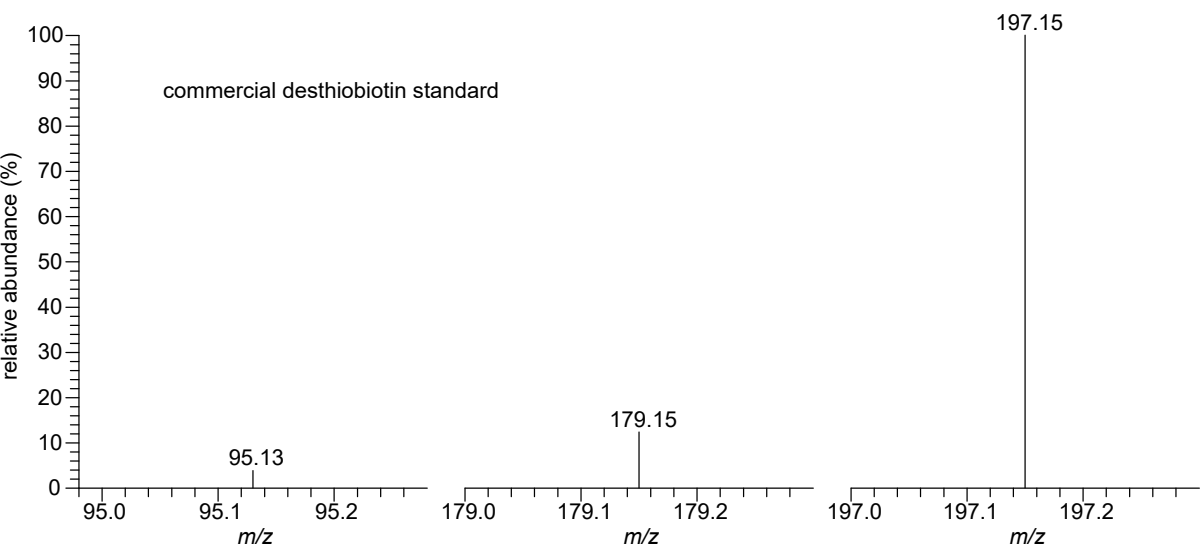

Supplement: Supplementary file 8 — Supplementary Figure 5 [file 41396_2022_1304_MOESM8_ESM.pdf]

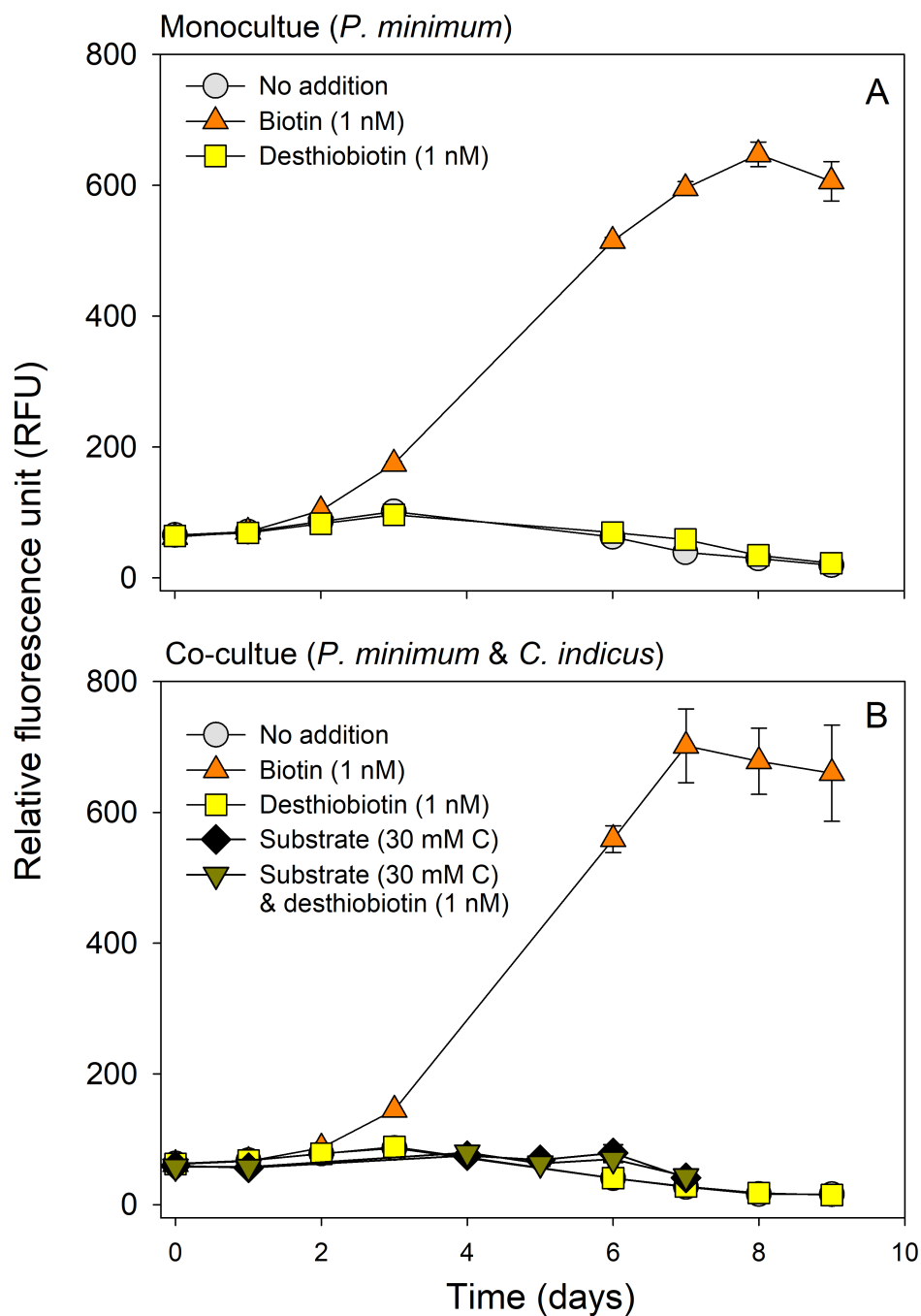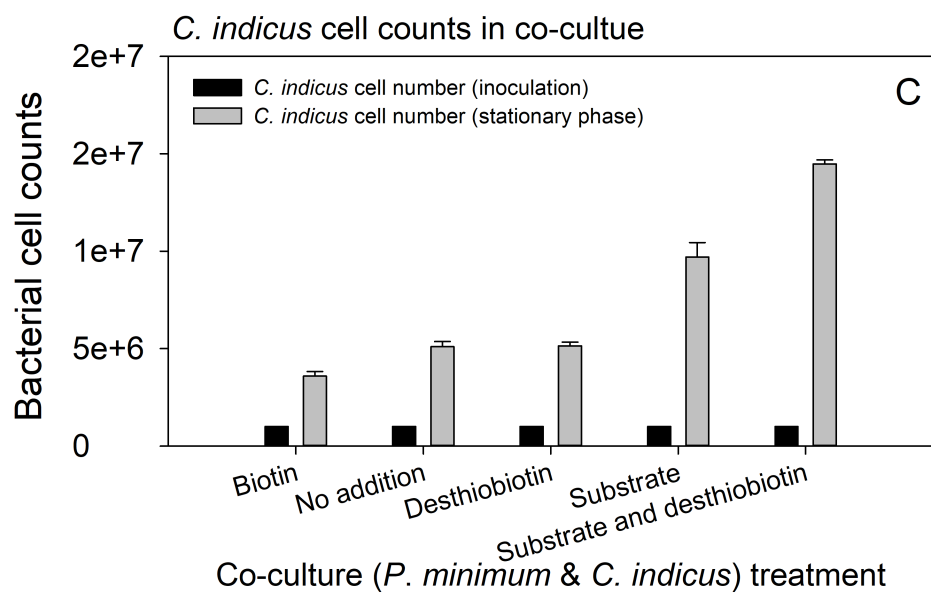

Supplement: Supplementary file 9 — Supplementary Figure 6 [file 41396_2022_1304_MOESM9_ESM.pdf]
