# Supplementary material for: The overlooked role of a biotin precursor for marine bacteria - desthiobiotin as an escape route for biotin auxotrophy
Source: ISME J. 2022 Aug 13;16(11):2599–609. doi: 10.1038/s41396-022-01304-w (PMC9561691; doi:10.1038/s41396-022-01304-w)
Supplement: Supplementary file 10 — Supplementary Data 1 [file 41396_2022_1304_MOESM10_ESM.docx]

**AB medium for 1L (Taga & Xavier (2011); with minor modifications,)**

NaCl……………………………………………17,5 g
MgSO4…………………………………………12,3 g
DI water………………………………..……….970 ml

- dissolve and bring to pH 7,5 with 3N NaOH (ca. 10ul NaOH)
- autoclave at 121°C and add after cooling down

Sterile stock solutions

1 M Potassium phosphate (pH 7)…………...10 ml
(use ca. 11 NaOH pellets to bring to pH 7)

Glutamate………………………………………2 mM (10 mMC)
